# Inflammatory and Neurotrophic Factors and Their Connection to Quality of Life in Progressive Supranuclear Palsy—Single-Center Study

**DOI:** 10.3390/ijms262412122

**Published:** 2025-12-17

**Authors:** Michał Markiewicz, Bartosz Migda, Dagmara Otto-Ślusarczyk, Natalia Madetko-Alster, Alicja Wiercińska-Drapało, Maciej Darewicz, Marta Struga, Piotr Alster

**Affiliations:** 1 Department of Neurology, Medical University of Warsaw, Kondratowicza 8, 03-242 Warsaw, Polandnatalia.madetko@wum.edu.pl (N.M.-A.); 2 Diagnostic Ultrasound Lab, Department of Pediatric Radiology, Medical University of Warsaw, 03-242 Warsaw, Poland; bartosz.migda@wum.edu.pl; 3 Department of Biochemistry, Medical University of Warsaw, Banacha 1, 02-097 Warsaw, Poland; dagmara.otto@wum.edu.pl (D.O.-Ś.); marta.struga@wum.edu.pl (M.S.); 4 Department of Hepatology and Infectious and Tropical Diseases, Medical University of Warsaw, Provincial Infectious Diseases Hospital in Warsaw, Wolska 37, 01-201 Warsaw, Poland; alicja.wiercinska-drapalo@wum.edu.pl (A.W.-D.); mdarewicz@mp.pl (M.D.)

**Keywords:** neurodegeneration, neuroinflammation, progressive supranuclear palsy (PSP), quality of life, QoL

## Abstract

Progressive supranuclear palsy (PSP) is a condition classified as atypical parkinsonism. Pathologically, it is a four-repeat tauopathy; clinically, it is a disease comprising oculomotor dysfunction, postural instability, akinesia, and cognitive/language disorders. Its pathogenesis is not fully recognized; however, neuroinflammation is considered to likely be a significant aspect, though it is not known whether inflammation is a cause or consequence of neurodegeneration. In this study, the authors analyzed the association between inflammatory/neurotrophic factors and parameters linked to quality of life, based on examinations of 10 controls and 11 patients with PSP. They found a negative correlation between mPSPRS (Modified Progressive Supranuclear Palsy Rating Scale) and the glial cell-line-derived neurotrophic factor levels (GDNF) (r = −0.772727, *p* = 0.003) in the serum, a less pronounced negative correlation between progressive supranuclear palsy–quality of life parameter (PSP-QoL) and GDNF (r = −0.68390, *p* = 0.011) in the serum, and no correlations were observed in the analyses of inflammatory factors. The obtained results show the association between lower levels of GDNF and more pronounced clinical deterioration. Further analysis in the field based on larger groups of patients is required.

## 1. Introduction

Progressive supranuclear palsy (PSP), the most common among the atypical parkinsonian disorders (Parkinson-plus syndromes), is a neurodegenerative disease with a largely unknown pathophysiological background [1]. Its clinical manifestation is associated with oculomotor dysfunction and speech/cognitive deterioration, differing from typical parkinsonian syndrome (tremor, akinesia, rigidity, and postural instability) [1]. Although the diagnostic criteria include multiple subtypes of PSP, the vast majority (up to 90% of all cases) are linked with two subtypes: PSP-Richardson’s syndrome (PSP-RS) and PSP-parkinsonism predominant (PSP-P), which have different clinical presentations and prognoses. The leading neuropathological process is the aggregation of microtubule-associated protein tau (MAPT) into neurofibrillary tangles, and it is present in both neurons and glia cells in several cortical and subcortical regions of the brain [2], with continuous accumulation resulting in microgliosis, astrogliosis, and neuronal damage [3]. Factors describing the pathogenesis of PSP include mitophagy disruption, oxidative stress, and neuroinflammation, among others [4]. Apart from the concepts of genetic and epigenetic background, and, recently, proteome-wide expression discoveries, there are convincing data for neuroinflammation’s involvement in PSP pathogenesis [5,6,7].

In several studies, including those conducted posthumously, increased microglia numbers and activation were identified in PSP brains, especially in the subthalamic nucleus, substantia nigra, extrapyramidal motor system, and its cerebellar output [8]. There was a positive correlation between microglial activation and tau concentration [9]. In PET imaging studies, microglial activation was identified in the midbrain, basal ganglia, and other areas crucial for PSP symptomatology [10]. Interestingly, tau accumulation and microglial activation were proven to be positively correlated with clinical course severity and were predictive factors for disease progression [11,12]. These results strongly support the hypothesis of microglial activation being a major pathway of PSP pathology. The results from transgenic animal studies on tau-overexpressed rats suggest that the primary cause of microglial activation is tau deposits, as inflammation starts in tangle areas; in vitro cultures are found to secrete pro-inflammatory cytokines in the presence of full-length tau structures [13]. Pro-inflammatory factor release and neuroinflammation induction further accelerate the hyperphosphorylated tau accumulation process, leading to a high concentration of pro-inflammatory factors such as IL-1β being identified in PSP pathology-related structures [8,14]. It is not clear whether neuroinflammation leads to neurodegeneration or whether it is rather a consequence of the degenerative process [15]. The other mechanism that is common for many neurodegenerative diseases is glial cell senescence, which directly contributes to neuronal tau pathology, resulting in cognitive impairment, although its significance in PSP pathology is unknown and there is currently limited evidence in this field [16]. Also, limited data are available regarding T cell involvement: studies on genetically modified rodents resulting in T cell infiltration suppression suggest its protective role in PSP pathophysiology; therefore, further research is needed [17]. Hepcidin is a peptide of liver origin, primarily identified as an iron homeostasis regulator, and its tissue distribution (including CNS) plays a role in neuroinflammation and the pathogenesis of neurodegenerative diseases; therefore, more data are available for Alzheimer’s disease and Parkinson’s disease, and its significance in atypical parkinsonism has been described less [18].

Recently, there has been growing interest in neurotrophin dysregulation and tauopathy development. Among others, brain-derived neurotrophic factor (BDNF) is identified as a major agent involved in such processes [19]. Its mRNA and protein concentrations are decreased in post mortem brain studies, followed by clinical decline [20,21]. Neurotrophin dysregulation by tau-mediated pathways seems to be commonly identified in various neurodegenerative disorders; nevertheless, in some studies, the nerve growth factor (NGF) binding site density was noticeably lower in PSP, but not in other parkinsonian syndromes [22]. To the best of our knowledge, the only study to extensively explore the significance of neurotrophins in PSP was performed by Alster et al. [23]; the results indicated potentially striking aspects regarding the pathophysiology of the disease. In clinical practice, measuring non-specific inflammatory factors’ concentrations from samples obtained invasively is impractical; therefore, the analysis of easily accessible factors seems feasible. Also, the link between laboratory features and clinical insight is crucial for current assessment and follow-up. Future diagnostic management will possibly include multimodal diagnostic algorithms integrating structured clinical phenotyping, quantitative imaging, molecular diagnostics, and, optimally, autopsy-linked validation [24]. Evaluating factors important for a patient’s everyday life functioning supports the patient-orientated perspective, so we performed our analysis based on quality of life (QoL) parameters.

## 2. Results

A total of 11 patients with PSP and 10 healthy controls were included in the study. Clinical severity in the PSP group was assessed using the mPSPRS and PSP-QoL scales, with mean values of 6.36 ± 4.23 and 106.73 ± 57.98, respectively.

### 2.1. Biomarker Concentrations in Serum and CSF

We first compared biomarker concentrations between PSP patients and healthy controls.

**Figure 1:**
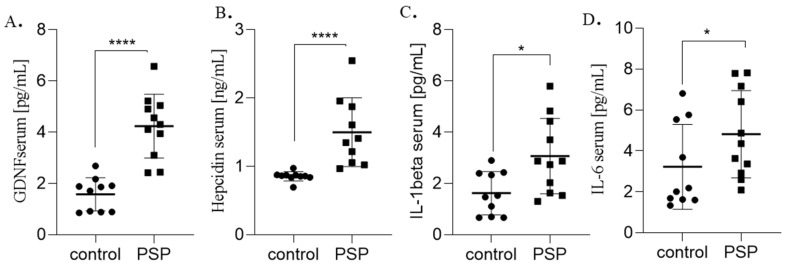
Serum biomarkers levels in PSP patients and healthy controls. Box-and-dot plot showing individual values, mean ± SD for serum (**A**) GDNF, (**B**) hepcidin, (**C**) IL-1 beta, (**D**) IL-6 concentrations in patients with PSP and healthy controls. A higher GDNF and IL-6 level was observed in the PSP group compared to controls (**** *p* = 0.0001, * *p* = 0.01, respectively, Mann–Whitney U test).

**Figure 2:**
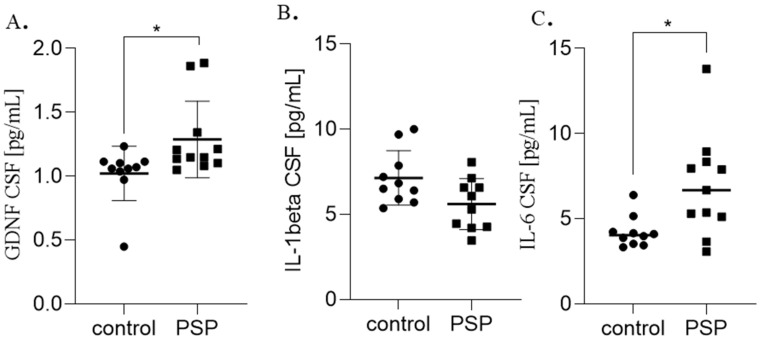
CSF biomarkers levels in PSP patients and healthy controls. Box-and-dot plot showing individual values, mean ± SD for serum (**A**) GDNF, (**B**) IL-1 beta, (**C**) IL-6 concentrations in patients with PSP and healthy controls. A significantly lower all biomarkers level was observed in the PSP group compared to controls (* *p* = 0.01, respectively, Mann–Whitney U test).

Significant group differences were observed for serum GDNF, hepcidin, IL-1β, and IL-6, as well as for CSF GDNF and IL-6 (Table 1). The distribution of serum biomarkers is shown in Figure 1 and CSF biomarkers in Figure 2.

### 2.2. Correlations Between Biomarkers and Clinical Scales

We next investigated associations between biomarker levels and clinical severity within the PSP group.

**Figure 3:**
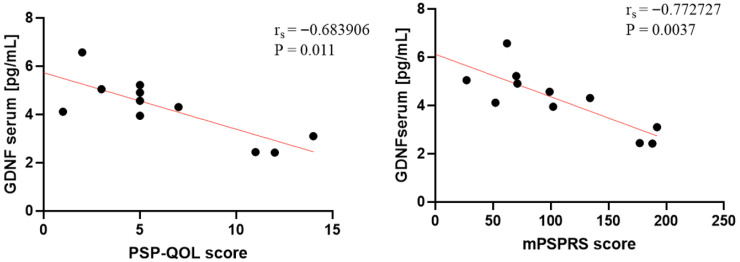
Correlation between clinical scales and biomarkers in PSP patients and healthy controls. Significant negative correlations were observed in the PSP group for mPSPRS (ρ = −0.77, *p* = 0.003), and PSP-QoL (ρ = −0.68, *p* = 0.01). Each panel shows individual data points (n = 11) with a regression line in red. PSP = progressive supranuclear palsy; GDNF = glial cell line-derived neurotrophic factor.

Significant negative correlations were observed between serum GDNF and both clinical scales: mPSPRS (ρ = −0.77, *p* = 0.003) and PSP-QoL (ρ = −0.68, *p* = 0.011). These relationships are illustrated in Figure 3 and summarized in Table 2.

No significant correlations were identified for IL-1β, IL-6, or hepcidin in either serum or CSF.

In healthy controls, a negative correlation between CSF IL-6 and mPSPRS was observed (ρ = −0.60, *p* = 0.036).

No other significant correlations were observed across the remaining markers and scales in either group.

## 3. Discussion

Although the etiology of PSP is not specified, in terms of its pathogenesis, inflammation and oxidative damage are thought to greatly contribute to disease development. In this study, we evaluate the neuroinflammation (expressed as the concentration of specific markers) and neurotrophic factors in connection with the functional status of PSP patients.

In patients with PSP, there was a significant correlation between serum GDNF and clinical-scale results: mPSPRS and PSP-QoL (*p* = 0.003, *p* = 0.01, respectively). This suggests that this molecule possibly plays a protective role in neuron survival. GDNF is significant as it is considered a crucial agent for dopaminergic cells preservation, although its role in neurodegenerative diseases is not fully recognized—in Parkinson’s disease, it favors dopaminergic neuron survival, and, additionally, the results of certain studies based on a limited cohort of PSP patients suggest that GDNF release may be a mechanism contrary to the neurodegeneration process in PSP-RS [23,25].

From a clinical perspective, the connection between GDNF changes and sleep disturbances [26], some executive functions (especially working memory, inhibitory control and cognitive flexibility) [23], and cognitive status [27] was proven in several works; in each, the protective role of this neurotrophin was highlighted. None of these papers evaluated the overall QoL, different tools to analyze functional decline were chosen, and some of them were based on examining conditions other than PSP, e.g., Parkinson’s disease; therefore, a general conclusion cannot be made.

Alster et al. [25] highlighted that this factor had a possibly protective role, especially in more deteriorating forms of PSP, and GDNF release was considered to potentially be a mechanism contrary to rapid neurodegeneration in PSP-RS. In this ante mortem study, it was found that more increased serum GDNF levels were observed in PSP-P, though this observation was not confirmed in PSP-RS, which suggests that serum GDNF levels may primarily increase as a protective mechanism; this hypothesis aligns with the results of GDNF studies in Parkinson’s disease, where the factor had an inhibitory influence on the deterioration of certain clinical features (discussed above). As more pronounced atrophy is generally observed in PSP-RS than PSP-P, this suggests that the rise in GDNF may be seen earlier in the disease course of the former than in the latter [28].

On the other hand, if GDNF release is a protective reaction to degeneration, its level may increase proportionally to the atrophy severity regardless of PSP clinical phenotype; due to the undefined and possibly multifactorial pathomechanism of this disease, presumptions such as this are limited. Further studies on PSP patients with a well-established disease subtype diagnosis, ideally with subsequent serial sampling of serum at different disease stages, would verify this prediction. In such a situation, GDNF evaluation could be used as a marker of future neuroprotective therapies’ effectiveness in various neurodegenerative diseases, yet further studies are needed to confirm its usefulness in such a role. The results of our research team demonstrate the connection between biochemical and clinical aspects of PSP, resembling similar neurodegenerative diseases like PD. In light of these similarities, the authors intend to enroll patients diagnosed with other atypical parkinsonian syndromes to explore the laboratory and clinical features of the disorders.

Significant differences in some interleukins’ (e.g., IL-1 and IL-6) concentrations with respect to different PSP subtypes were identified in a recent study [29]. The authors of that study differentiated between PSP-P and PSP-RS patients and explained several interleukin patterns in specific microglial activity leading to various types of IL release; they also assumed the potential impact of these immune profiles on the natural course of the disease. As in our study, there was only a trend toward significance in terms of IL-1β concentration, so we assume that enrolling a larger cohort (or multi-center data) would make the sample strong enough to achieve statistical significance in studies of that strongly pro-inflammatory cytokine. This is because other animal studies have revealed its role in various degenerative processes in the central nervous system, where the neuroprotective effect of microglia activation via IL-1β pathways was identified [30]. In a study on the mechanisms of microglia activation via the IL-6 pathway in traumatic brain injury, the authors achieved similar results; therefore, cases of severe CNS injury should be included [31].

Preclinical studies on inflammasome activation reveal that in the production of pro-inflammatory cytokines IL-1β and IL-18, tau hyperphosphorylation and accumulation are increased [32]. We assume that further clinical studies on IL-18 in PSP would provide coherent results. The preclinical findings highlight that the inflammasome may be a therapeutic target in PSP and other tauopathies [33].

Apart from more specific cytokines, non-specific peripheral inflammatory parameters were also investigated in PSP and other parkinsonian-plus syndromes. As the inflammatory process in neurodegenerative diseases is generalized, it can be reflected by non-specific parameters. Studies on the serum lymphocyte-to-monocyte ratio (LMR), neutrophil-to-high-density-lipoprotein ratio (NHR), and neutrophil-to-lymphocyte ratio (NLR) revealed elevated NLR and NHR parameters with lower LMR in patients with PD, corresponding with the disease severity; the platelet-to-lymphocyte ratio (PLR) was significantly lower in PD, but not in other parkinsonian syndromes (as MSA and PSP patients were analyzed) [34]. The explanation for these observations remains unclear; therefore, it is suggested that different pathomechanisms are involved in these disorders. As discussed, the parameters are highly unspecific, and the impacts of general patient status and numerous other parameters need to be ruled out.

On the other hand, studies have revealed substantially increased GDNF levels in PSP-P patients and less increased levels in PSP-RS patients; the authors suggest that in PSP, the GDNF levels in the serum initially rise as a protective mechanism [29]. Such a mechanism is considered for dopaminergic neurons protection in PD, a disorder where more data is available, compared to Parkinson-plus syndromes [23]. In PD animal models, a positive effect on various glial cells was observed; this could be a novel therapeutic strategy for such neurodegenerative disease, with potential for similar disorders [23]. Preclinical findings on GDNF’s role include promoting the survival of remaining neurons and axons regeneration as well as the promotion of the functional activity of viable neurons [35].

Also, higher hepcidin levels were identified in the serum in PSP-RS [18]. Furthermore, the inclusion of other rare PSP subtypes could be beneficial for exploring this hypothesis, but obtaining results of statistical significance might be challenging due to cohorts probably being small. In further studies, the analysis of comorbidities and their influence on neurotrophic agent concentrations could also bring innovative conclusions.

In studies of small patient cohorts, strong microglial activation and its correlation with tau accumulation were found in both –PSP and corticobasal degeneration (CBD) on brain sections, although there were no clinical (or QoL) analyses of those few cases (for PSP, n = 10) [9]. Also, in larger groups with 95 PSP and 30 CBD cases, it was confirmed at autopsy that they had identified microscopic findings, but without any connection to clinical symptoms and without an impact on QoL [36]. Inconclusive results for the wider immune profile in PSP (for interleukin 4, tumor necrosis factor α [TNF-α], and transforming growth factor β1 [TGF-β1] analysis) suggest that only a few molecules could be specific markers for neuroinflammation [37]. Promising results (cytokine concentration in PSP in comparison to PD) come from studies on interleukin 10, interleukin 18, interleukin 1β, and interferon γ [38]. A meta-analysis of 29 studies on a total of 1679 patients revealed poor correlation of central with peripheral inflammatory markers [39,40]. The imbalance of pro- and anti-inflammatory homeostasis leads to clinical symptoms that influence patient well-being and disease burden on everyday life, decreasing QoL. As PSP seems to be heterogeneous, further studies on the disease pathophysiology and clinical presentation should be performed with an acknowledgement of its major subtypes. For future studies, in addition to developing our pilot study outcomes, an attempt at a clinical distinction of PSP-P and PSP-RS based on QoL evaluation results should be made.

It is noteworthy that the intervention based on modifying the disease course only via central inflammation suppression is unlikely to stop the pathological process and could therefore delay the clinical deterioration of some patients. The GDNF and its pathway seem to be a promising aim, as they were identified as influencing numerous clinical symptoms of PSP; nevertheless, more real-life data (including QoL studies) are needed.

As in PSP-RS, with clinically relatively rapid deterioration, the most intense inflammatory process is present at the beginning of the disease; the studies revealed higher inflammatory marker concentrations at the beginning of the course. In PSP-P, inflammatory activity seems to be less intense and stable; therefore, the concentrations of pro-inflammatory cytokines are predicted to be lower than in RS cases and stable in subsequent samples. This hypothesis based on clinical observations of the natural PSP course requires further studies focused on changes in interleukin concentrations during the following disease stages.

### Limitations

This study has several limitations. Firstly, the number of examined patients and proportionally healthy controls was limited (reflecting a low morbidity and relatively short disease duration), as the research was planned as a pilot single-center study. Given the pilot nature and small sample size, the findings should be considered preliminary.

Limited group size has an influence on limitations in statistical methodology of the study. No formal multiple-comparison adjustments were applied and the results should be interpreted as preliminary. Future studies with larger sample sizes will incorporate appropriate statistical procedures, including false discovery rate (FDR) correction, among others.

Importantly, in the analysis there was no distinction between PSP-RS and PSP-P subtypes. Similar to work by Alster et al. [18], the results are presumably the outcome of an even smaller group of patients that are potentially more heterogeneous (including various disease phenotypes). In that study, serum GDNF levels increased in both PSP subgroups when compared to the controls, which supports the hypothesis that in all cases of PSP, the serum levels are higher than in HCs. In PSP-RS, the concentration in the CSF was significantly increased compared to PSP-P or HCs (and for both of those cohorts, the concentration was comparable). To the best of our knowledge, there are no studies on GDNF concentration in PSP variants other than –RS or –P.

The clinical deterioration observed in PSP limits the possibility of extended follow-up evaluation, and also leads to bounded cooperation of patients in research projects of observational protocol only. As a result of this fact, longitudinal data (including subsequent sampling of the material obtained in an invasive way) was not available. Studies based as a development of this work would contribute novel data, including the dynamic changes in indicators in the disease progression timeline.

Although the authors intended to enroll healthy volunteers of similar age to the patients, some of them were also diagnosed with chronic disorders (e.g., cardiovascular diseases or joint degeneration) that can influence QoL outcomes. However, the detailed analysis of available medical history of all participants of the study did not reveal facts substantially affecting their status.

The patients’ clinical diagnoses were not verified neuropathologically; therefore, obtaining a definite diagnosis was not accessible. The study is based on probable/possible diagnosis of PSP. All of the patients were examined by neurologists who are experienced in movement disorders.

As each of the participants was evaluated once, it was not possible to identify any changes in studied values over time, though non-specific peripheral inflammation parameters are known to be variable. Serum and CSF samples were taken before the QoL interview, and the interval was not standardized; the authors plan to analyze the influence of this delay on results in further studies. This makes it difficult to clarify the correlation between the trends in factors such as GDNF and the disease course. Several improvements are considered for future study: longitudinal follow-up, with multi-time biomarker testing and clinical assessments, is planned, which could possibly allow for dynamic changes in indicators to be tracked as the disease progresses.

## 4. Materials and Methods

### 4.1. Group Description and Examination Methods

In this study, we analyzed biochemical parameters in the serum and cerebrospinal fluid (CSF) of patients diagnosed with PSP in comparison to healthy controls (HCs) of a similar age. The mean age in the PSP group was 70.5 years (SD = 5.35), ranging from 60 to 81; in the HC group, it was 66.3 years (SD = 5.73), ranging from 59 to 78. Gender distribution showed a noticeable male predominance in the PSP group alone: 63.6% male in the PSP group (4 females, 7 males) and 70.0% female (7 females, 3 males) in the HC group. The disease duration ranged between 3 and 6 years.

All patients in the study were diagnosed in the Department of Neurology of the Medical University of Warsaw based on the current diagnostic criteria by neurologists highly experienced in movement disorders [1], with written consent provided by all participants. Exclusion criteria were the diagnosis of neoplasmatic diseases, autoimmune diseases, infectious diseases, hematologic diseases, diabetes or metabolic syndrome, and using drugs with a possible impact on the analyzed factors; none of the patients used medication that significantly impacted the inflammatory parameters. All participants underwent morphological examination and lumbar puncture in the Department of Neurology of the Medical University of Warsaw, and serum and CSF biochemical examinations were conducted in the Department of Biochemistry. From each patient, 10 mL of CSF and 10 mL of serum were taken for analysis, frozen at −80 °C soon after the collection procedure, and stored in such conditions until evaluation. Each sample was analyzed regarding the four parameters—interleukin 1 β (IL-1β), interleukin 6 (IL-6), hepcidin, and glial cell line neurotrophic factor (GDNF). Commercial ELISA kits from Diaclon SAS (Besançon, France) were used for GDNF concentration assessment (absorbance determination at 450 nm with a plate reader) and calculation was conducted based on standard curves. For each parameter, doubled analysis was performed. The modified PSP Rating Scale (mPSPRS) and PSP-QoL were completed at least 1 week after discharge from the hospital, with caregiver support if necessary.

### 4.2. Statistical Analysis

All statistical analyses were performed using GraphPad Prism (version 8, GraphPad Software, San Diego, CA, USA) and variables were assessed for normality using the Shapiro-Wilk test. Given the small sample size and non-normal data distribution, non-parametric tests were applied throughout the analysis.

For group comparisons between PSP patients (n = 11) and healthy controls (n = 10), the Mann–Whitney U test was used to evaluate differences in serum and CSF biomarker levels, including GDNF, hepcidin, IL-1β, and IL-6.

To investigate the relationship between clinical scale scores and biomarker concentrations within the PSP group, Spearman’s rank correlation coefficients (ρ) were calculated. Clinical scales included the modified PSP Rating Scale (mPSPRS) and PSP-QoL, with statistical significance defined as *p* < 0.05. Spearman’s rank correlation was used to explore associations between biomarker concentrations in serum and cerebrospinal fluid (CSF) and clinical scale scores (mPSPRS and PSP-QoL) in both PSP patients and HCs.

## 5. Conclusions

Further research in neurodegenerative diseases needs to be focused on understanding the role of inflammation as a possibly significant pathogenic factor [33]. So far, none of therapeutic interventions evaluated in PSP have been proven to have sufficient effectiveness, despite over 30 drugs having been tested; the molecules’ targets were designed based on discussions of pathophysiology (mostly tau aggregation and accumulation slow-down or microglia activation inhibition) [41]. For innovative therapies, achieving both laboratory and clinical end-points is necessary. Using QoL to indirectly assess clinical outcomes seems to be promising. Future therapies based on neurotrophic agent interference are possibly promising in the context of current therapeutic nihilism regarding atypical parkinsonian syndromes [42].

Inflammation and deviations in neurotrophic parameters play a role in PSP pathophysiology and similar degenerative diseases. In our study, where neuroinflammation corresponds with symptoms and patient functional status, we underline the importance of preclinical and clinical features, encouraging future multi-center and multi-perspective research regarding PSP and related disorders to be conducted.

## Figures and Tables

**Table 1:** Comparison of biomarker concentrations in serum and CSF between PSP patients and healthy controls using the Mann–Whitney U test.

Biomarker	Control (Means ± SD)	PSP (Mean ± SD)	*p*-Value
**Serum**			
GDNF pg/mL	1.60 ± 0.6	2.23 ± 1.2	**** *p* = 0.0001
Hepcidin ng/mL	0.85 ± 0.1	1.88 ± 1.3	**** *p* = 0.0001
IL-1 beta pg/mL	1.62 ± 0.8	3.06 ± 1.5	* *p* = 0.01
IL-6 pg/mL	3.72 ± 2.2	4.81 ± 2.1	* *p* = 0.04
**CSF**			
GDNF pg/mL	1.02 ± 0.2	1.28 ± 0.3	* *p* = 0.01
IL-1 beta pg/mL	7.14 ± 1.6	6.19 ± 1.5	*p* = 0.07
IL-6 pg/mL	4.21 ± 0.9	6.92 ± 2.9	* *p* = 0.01

* *p* < 0.05, **** *p* < 0.0001 (Mann–Whitney U test).

**Table 2:** Spearman’s correlations between clinical scale scores and serum biomarker concentrations in PSP patients.

Group	Clinical Scale	Biomarker	Specimen	Spearman’s r	*p*-Value
PSP	mPSPRS	GDNF	Serum	**−0.772727**	** *p* = 0.003
PSP	PSP-QoL	GDNF	Serum	**−0.683906**	* *p* = 0.01

* *p* < 0.05, ** *p* < 0.01. Spearman’s rank correlation was used. Trends toward significance are noted where *p* < 0.1.

## Data Availability

The original contributions presented in this study are included in the article. Further inquiries can be directed to the corresponding author.

## References

[1] Höglinger G.U., Respondek G., Stamelou M., Kurz C., Josephs K.A., Lang A.E., Mollenhauer B., Müller U., Nilsson C., Whitwell J.L. (2017). Clinical diagnosis of progressive supranuclear palsy: The movement disorder society criteria. Mov. Disord. Off. J. Mov. Disord. Soc..

[2] Hauw J.J., Daniel S.E., Dickson D., Horoupian D.S., Jellinger K., Lantos P.L., McKee A., Tabaton M., Litvan I. (1994). Preliminary NINDS neuropathologic criteria for Steele-Richardson-Olszewski syndrome (progressive supranuclear palsy). Neurology.

[3] Boxer A.L., Yu J.-T., Golbe L.I., Litvan I., Lang A.E., Höglinger G.U. (2017). Advances in progressive supranuclear palsy: New diagnostic criteria, biomarkers, and therapeutic approaches. Lancet Neurol..

[4] DeRosier F., Hibbs C., Alessi K., Padda I., Rodriguez J., Pradeep S., Parmar M.S. (2024). Progressive supranuclear palsy: Neuropathology, clinical presentation, diagnostic challenges, management, and emerging therapies. Dis. Mon..

[5] Fołta J., Rzepka Z., Wrzesniok D. (2025). The Role of Inflammation in Neurodegenerative Diseases: Parkinson’s Disease, Alzheimer’s Disease, and Multiple Sclerosis. Int. J. Mol. Sci..

[6] Dick F., Johanson G.A.S., Tysnes O.B., Alves G., Dölle C., Tzoulis C. (2025). Brain Proteome Profiling Reveals Common and Divergent Sig-natures in Parkinson’s Disease, Multiple System Atrophy, and Progressive Supranuclear Palsy. Mol. Neurobiol..

[7] Block M.L., Hong J.S. (2005). Microglia and inflammation-mediated neuro-degeneration: Multiple triggers with a common mechanism. Prog. Neurobiol..

[8] Fernandez-Botran R., Ahmed Z., Crespo F.A., Gatenbee C., Gonzalez J., Dickson D.W., Litvan I. (2011). Cytokine expression and microglial activation in progressive supranuclear palsy. Park. Relat. Disord..

[9] Ishizawa K., Dickson D.W. (2001). Microglial activation parallels system degeneration in progressive supranuclear palsy and cor-ticobasal degeneration. J. Neuropathol. Exp. Neurol..

[10] Malpetti M., Passamonti L., Rittman T., Jones P.S., Rodríguez P.V., Bevan-Jones W.R., Hong Y.T., Fryer T.D., Aigbirhio F.I., O’Brien J.T. (2020). Neuroinflammation and tau colocalize in vivo in progressive supranuclear palsy. Ann. Neurol..

[11] Malpetti M., Passamonti L., Jones P.S., Street D., Rittman T., Fryer T.D., Hong Y.T., Vàsquez Rodriguez P., Bevan-Jones W.R., Aigbirhio F.I. (2021). Neuroinflammation predicts disease progression in progressive supranuclear palsy. J. Neurol. Neurosurg. Psychiatry.

[12] Palleis C., Sauerbeck J., Beyer L., Harris S., Schmitt J., Morenas-Rodriguez E., Finze A., Nitschmann A., Ruch-Rubinstein F., Eckenweber F. (2021). In vivo assessment of neuroinflammation in 4-repeat tauopathies. Mov. Disord..

[13] Morales I., Jimenez J.M., Mancilla M., Maccioni R.B. (2013). Tau oligomers and fibrils induce activation of microglial cells. J. Alz-Heimers Dis..

[14] Gorlovoy P., Larionov S., Pham T.T., Neumann H. (2009). Accumulation of tau induced in neurites by microglial proinflammatory mediators. FASEB J..

[15] Koziorowski D., Figura M., Milanowski Ł.M., Szlufik S., Alster P., Madetko N., Friedman A. (2021). Mechanisms of Neurodegener-ation in Various Forms of Parkinsonism-Similarities and Differences. Cells.

[16] Si Z., Sun L., Wang X. (2021). Evidence and perspectives of cell senescence in neurodegenerative diseases. Biomed. Pharmacother..

[17] Laurent C., Dorothée G., Hunot S., Martin E., Monnet Y., Duchamp M., Dong Y., Légeron F.-P., Leboucher A., Burnouf S. (2017). Hippocampal T cell infiltration promotes neuroinflammation and cognitive decline in a mouse model of tauopathy. Brain.

[18] Alster P., Otto-Ślusarczyk D., Wiercińska-Drapało A., Struga M., Madetko-Alster N. (2024). The potential significance of hepcidin evaluation in progressive supranuclear palsy. Brain Behav..

[19] Mufson E.J., Counts S.E., Fahnestock M., Ginsberg S.D. (2007). Cholinotrophic molecular substrates of mild cognitive impair-ment in the elderly. Curr. Alzheimer Res..

[20] Peng S., Wuu J., Mufson E.J., Fahnestock M. (2005). Precursor form of brain-derived neurotrophic factor and mature brain-derived neurotrophic factor are decreased in the pre-clinical stages of Alzheimer’s disease. J. Neurochem..

[21] Fahnestock M., Michalski B., Xu B., Coughlin M.D. (2001). The precursor pro-nerve growth factor is the predominant form of nerve growth factor in brain and is increased in Alzheimer’s disease. Mol. Cell. Neurosci..

[22] Villares J., Strada O., Faucheux B., Javoy-Agid F., Agid Y., Hirsch E.C. (1994). Loss of striatal high affinity NGF binding sites in progres-sive supranuclear palsy but not in Parkinson’s disease. Neurosci. Lett..

[23] Tong S.Y., Wang R.-W., Li Q., Liu Y., Yao X.-Y., Geng D.-Q., Gao D.-S., Ren C. (2023). Serum glial cell line-derived neurotrophic factor (GDNF) a potential biomarker of executive function in Parkinson’s disease. Front. Neurosci..

[24] Arias-Carrión O., Romero-Gutiérrez E., Ortega-Robles E. (2025). Toward Biology-Driven Diagnosis of Atypical Parkinsonian Disorders. NeuroSci.

[25] Alster P., Otto-Ślusarczyk D., Szlufik S., Duszyńska-Wąs K., Drzewińska A., Wiercińska-Drapało A., Struga M., Kutyłowski M., Friedman A., Madetko-Alster N. (2024). The significance of glial cell line-derived neurotrophic factor analysis in Progressive Supranuclear Palsy. Sci. Rep..

[26] Wang L., Gao Z., Chen G., Geng D., Gao D. (2023). Low levels of adenosine and GDNF are potential risk factors for Parkinson’s disease with sleep disorders. Brain Sci..

[27] Numakawa T., Kajihara R. (2023). Neurotrophins and other growth factors in the pathogenesis of Alzheimer’s disease. Life.

[28] Alster P., Madetko N., Koziorowski D., Friedman A. (2020). Progressive Supranuclear Palsy-parkinsonism predominant (PSP-P)-A clinical challenge at the boundaries of PSP and parkinson’s disease (PD). Front. Neurol..

[29] Madetko-Alster N., Otto-Slusarczyk D., Wiercinska-Drapało A., Koziorowski D., Szlufik S., Samborska-Cwik J., Struga M., Friedman A., Alster P. (2023). Clinical Phenotypes of Progressive Supranuclear Palsy-The Differences in Interleukin Patterns. Int. J. Mol. Sci..

[30] Todd L., Palazzo I., Suarez L., Liu X., Volkov L., Hoang T.V., Campbell W.A., Blackshaw S., Quan N., Fischer A.J. (2019). Reactive microglia and IL1/IL-1R1-signaling mediate neuroprotection in excitotoxin-damaged mouse retina. J. Neuroinflamm..

[31] Willis E.F., MacDonald K.P.A., Nguyen Q.H., Garrido A.L., Gillespie E.R., Harley S.B.R., Bartlett P.F., Schroder W.A., Yates A.G., Anthony D.C. (2020). Repopulating Microglia Promote Brain Repair in an IL-6-Dependent Manner. Cell.

[32] Song L., Pei L., Yao S., Wu Y., Shang Y. (2017). NLRP3 Inflammasome in Neurological Diseases, from Functions to Therapies. Front. Cell. Neurosci..

[33] Gao C., Jiang J., Tan Y., Chen S. (2023). Microglia in neurodegenerative diseases: Mechanism and potential therapeutic targets. Signal Transduct. Target. Ther..

[34] Madetko N., Migda B., Alster P., Turski P., Koziorowski D., Friedman A. (2022). Platelet-to-lymphocyte ratio and neutrophilto-lymphocyte ratio may reflect differences in PD and MSA-P neuroinflammation patterns. Neurol. Neurochir. Pol..

[35] Sidorova Y.A., Saarma M. (2020). Small Molecules and Peptides Targeting Glial Cell Line-Derived Neurotrophic Factor Receptors for the Treatment of Neurodegeneration. Int. J. Mol. Sci..

[36] Yoshida M. (2014). Astrocytic inclusions in progressive supranuclear palsy and corticobasal degeneration. Neuropathology.

[37] Muñoz-Delgado L., Luque-Ambrosiani A., Zamora B.B., Macías-García D., Jesús S., Adarmes-Gómez A., Ojeda-Lepe E., Car-rillo F., Mir P. (2024). Peripheral immune profile and neutrophil-to-lymphocyte ratio in progressive supranuclear palsy: Case-control study and meta-analysis. Eur. J. Neurol..

[38] Hall S., Janelidze S., Surova Y., Widner H., Zetterberg H., Hansson O. (2018). Cerebrospinal fluid concentrations of inflammatory markers in Parkinson’s disease and atypical parkinsonian disorders. Sci. Rep..

[39] Gigase F.A.J., Smith E., Collins B., Moore K., Snijders G.J.L.J., Katz D., Bergink V., Perez-Rodriquez M.M., De Witte L.D. (2023). The association between inflammatory markers in blood and cerebrospinal fluid: A systematic review and meta-analysis. Mol. Psychiatry.

[40] Hartikainen P., Reinikainen K.J., Soininen H., Sirviö J., Soikkeli R., Riekkinen P.J. (1992). Neurochemical markers in the cerebrospinal fluid of patients with Alzheimer’s disease, Parkinson’s disease and amyotrophic lateral sclerosis and normal controls. J. Neural Transm. Park. Dis. Dement. Sect..

[41] Imbimbo B.P., Ippati S., Watling M., Balducci C. (2022). A critical appraisal of tautargeting therapies for primary and secondary tauopathies. Alzheimers Dement..

[42] Allen S.J., Watson J.J., Shoemark D.K., Barua N.U., Patel N.K. (2013). GDNF, NGF and BDNF as therapeutic options for neurodegeneration. Pharmacol. Ther..

